# Movement observation activates motor cortex in fibromyalgia patients: a fNIRS study

**DOI:** 10.1038/s41598-022-08578-2

**Published:** 2022-03-18

**Authors:** Eleonora Gentile, Antonio Brunetti, Katia Ricci, Vitoantonio Bevilacqua, Laila Craighero, Marina de Tommaso

**Affiliations:** 1 Applied Neurophysiology and Pain Unit, SMBNOS Department, Bari Aldo Moro University, Polyclinic General Hospital, 70136 Bari, Italy; 2 Department of Electrical and Information Engineering, Polytechnic University of Bari, 70126 Bari, Italy; 3 Department of Neuroscience and Rehabilitation, University of Ferrara, 44121 Ferrara, Italy

**Keywords:** Cognitive neuroscience, Sensorimotor processing

## Abstract

Scientific evidence points to a shared neural representation between performing and observing an action. The action observation notoriously determines a modulation of the observer’s sensorimotor system, a phenomenon called Motor Resonance (MR). Fibromyalgia (FM) patients suffer from a condition characterized by generalized musculoskeletal pain in which even simple movement can exacerbate their symptoms. Maladaptive functioning of the primary motor cortex is a common finding in patients with chronic pain. Activation of the motor cortex is known to induce an analgesic effect in patients with chronic pain. In this exploratory study, we intend to verify if the mere observation of a movement could elicit activation of the motor cortical areas in patients with FM. Therefore, the purpose of this study was to examine the presence of MR in patients affected by fibromyalgia. We adopted a behavioral paradigm known for detecting the presence of MR and a neurophysiological experiment. Participants watched videos showing gripping movements towards a graspable or an ungraspable object, respectively, and were asked to press a button the instant the agent touched the object (Time-to-contact detection session). In a different experimental session, participants were only requested to observe and pay attention to the videos (Observation-only session). During each experimental session, the participants’ cerebral hemodynamic activity was recorded using the functional Near-Infrared Spectroscopy method. The behavioral task analysis revealed the presence of MR in both FM patients and healthy controls. Moreover, neurophysiological findings suggested that the observation of movement during the Observation-only session provoked activation and modulation of the cortical motor networks of FM patients. These results could represent evidence of the possible beneficial effects of movement observation in restarting motor activation, notoriously reduced, in FM patients.

## Introduction

Experimental evidence indicates the presence of a representational sharing between the execution of an action and its observation^1^. An impressive body of functional magnetic resonance imaging^2^, magnetoencephalography^3^, electroencephalography^4^, and transcranial magnetic stimulation^5^ studies demonstrated the overlap of the neural networks associated with the perception and execution of action. One of the consequences of this overlap is that the perception of others’ actions is accompanied by a modulation of the observer’s corticospinal system. The term motor resonance specifically refers to this modulation characterized by somatotopic specificity (i.e., the pattern of muscle activation is similar to that of the agent) and high temporal fidelity (i.e., muscles activation is temporally coupled with the dynamics of the observed action), indicating that the perceived action is subliminally re-enacted^6–8^.

This development of our knowledge in basic neuroscience led to a rehabilitation approach called Action Observation Treatment (AOT)^9^, which aims to activate the motor cortices in patients with motor impairments. It takes advantage of the evidence that while observing other people performing daily actions, the neural structures involved in the actual execution of those actions are recruited as if the observers were performing that action. AOT has been used in the rehabilitation of patients suffering from chronic ischemic stroke^10^, in Parkinson’s disease patients^11^, and in children with cerebral palsy^12^. Interestingly, this treatment has also been used in patients with post-surgical pain or chronic pain^13^. Maladaptive plasticity of the primary motor cortex (M1) is a common finding in patients with chronic pain, specifically in patients with fibromyalgia (FM)^14,15^, a widespread chronic pain disease characterized by generalized musculoskeletal pain, fatigue, sleep disturbance and memory impairment, whose etiopathogenetic mechanisms are not yet known^16^. It has been proposed that M1 acts as a modulator of pain processing and that its reduced function could abnormally enhance response to sensory stimuli^14,15^ and change pain perception and processing in fibromyalgia^17^. Many studies have shown that modulation of the activity of the primary motor cortex (M1) induces analgesic effects in FM patients^18,19^. It is to note that FM patients have a peculiar limitation of movement^20,21^, and are unlikely to exercise because they fear the worsening of their painful condition^22^. Therefore, AOT is a good candidate as an alternative strategy for activating the motor cortex^23^ and its consequent analgesic effect^24^.

However, a series of experimental pieces of evidence suggested that the involvement of the motor system during the perception of others’ actions also depends on aspects not simply related to the kinematics of the movement, indicating the presence of a top-down modulation of motor resonance according to stimulus features and task requirements^25^. Specifically, motor facilitation was canceled in the presence of a mismatch between kinematics and explicit semantic clues relating to the action^26^, or situational contexts^27^, or intrinsic properties of the to-be-grasped object^28^.

Indeed, behavioral evidence showed that pain leads to reduced motor processing of others’ behavior^29^, and a recent systematic review and meta-analysis about the effects of AOT regarding the pain intensity in patients with musculoskeletal pain was unable to evaluate treatment efficacy due to very low-quality evidence^13^. Therefore, the theoretical question concerns the presence of motor resonance in patients with fibromyalgia, and the consequent clinical question regards the opportunity to apply AOT in these patients.

The aim of the present study was to investigate the presence of motor resonance in FM patients, and to compare the cerebral hemodynamic activity, detecting oxyhemoglobin and deoxyhemoglobin variations, of FM patients and healthy controls during action observation.

To this purpose, FM patients and matched controls were presented with videos showing grasping movements and, in different sessions, they were requested to just observe the videos, or to detect the contact time between the hand and the object. In half of the trials, by means of a graphic software, the kinematics of the videos was kept unchanged and the object replaced with an ungraspable one (i.e., the to-be-grasped bar was replaced by a bar of the same size but with sharp tips at the fingers opposition space). The instant in which the hand touched the object in the different videos was always the same. The authors capitalized on a robust paradigm from the same laboratory^28^ proved to be capable of providing neurophysiological and behavioral indices of the presence of motor resonance. Specifically, corticospinal activation was present only when the observed movement was suitable to grasp the object; moreover, in the same trials the detection times (i.e., responses to the contact time between the hand and the object) were more accurate^28^. These data, both neurophysiological and behavioral, indicate that motor resonance depends on the observer’s sensorimotor knowledge. Such knowledge, forged by experience, allows the observer to subliminally re-enact only suitable actions. Therefore, it is assumed that a modulation of detection times in this experimental paradigm is an index of the presence of motor resonance^28,30^. The absence of modulation of the detection time would indicate that the task would be performed without involving the sensorimotor system, and therefore no motor resonance would be present. The only difference between the experimental protocol used in the present experiment and that of Craighero et al.^28^ was that Transcranial Magnetic Stimulation was not used but functional Near-Infrared Spectroscopy.

During both experimental sessions (i.e., observation only and time-to-contact detection) the hemodynamic activity was recorded by functional Near-Infrared Spectroscopy (fNIRS). fNIRS is an established optical imaging method that uses near-infrared light to noninvasively quantify the hemodynamic responses associated with neural activity. The increased blood flow evoked by neural activity in a brain region usually results in an increase in oxyhemoglobin (HbO_2_) and a decrease in deoxyhemoglobin (HbR). Thanks to its low cost, good temporal resolution, portability, and movement tolerance, the fNIRS is well suited to study the activation in specific motor-related areas. Previous studies in FM patients^31^ showed that the motor cortex exhibits reduced levels of oxyhemoglobin during the resting state. These data encouraged our investigation as they suggest that patients could have a beneficial effect in action observation and motor cortex activation promotion.

In summary, we expected the control subjects to show faster responses during graspable object videos as compared to ungraspable object videos (i.e., motor facilitation, an index of motor resonance), confirming previous results^28,30^. A similar pattern in the responses of FM patients would indicate the presence of a normal motor resonance. Regarding the fNIRS data, we expected to find a difference in the hemodynamic activity between trials showing graspable and ungraspable object videos but only in those experimental conditions in which the modulation of the detection times indicated the presence of motor resonance. Finally, we decided to subject the participants to an observation session to test the influences on cortical metabolism of a situation as similar as possible to that of AOT. Furthermore, given that previous observation showed reduced metabolism of the motor cortical network during active movement^32^, we were interested in whether motor activation was greater when the motor response was required or when it was not, a valuable information for planning a rehabilitation treatment.

## Materials and methods

### Participants

Twenty-two FM patients (19 females, 50.45 ± 10.67 years) and twenty healthy adults (13 females, 46.30 ± 11.48 years) were enrolled in this experiment. FM patients were outpatients at the Applied Neurophysiology and Pain Center of Bari Polyclinic General Hospital, and they were recruited after their first access, before starting the pharmacological treatment. Diagnosis of FM was in accord with ACR criteria (2010). All the participants were right-handed according to the score of the Edinburgh Handedness Inventory^1^. The Ethics Committee of the Bari Polyclinic General Hospital approved the experimental design of the study. Each participant signed informed consent to participate in the experimental study. The experiment was performed in accordance with relevant guidelines and regulations. Exclusion criteria for each group were: less than 8 years of education, any peripheral or Central Nervous System (CNS) diseases not related to FM, including spinal cord diseases and radiculopathies, psychiatric diseases, diabetes, active and/or positive history for thyroid insufficiency, renal failure, auto-immune diseases, inflammatory arthritis, systemic connective tissue disease, present or previous history of cancer, as well as current use of drugs acting on the CNS or chronic opioid therapy.

### Probe placement and fNIRS system

The experiment was conducted with the continuous-wave NIRS system (NIRSport 8 × 8, Nirx Medical Technologies LLC, Berlin, Germany). It is a wearable, multi-channel, fNIRS device for the measurement of brain oxygenation. The fNIRS acquisition software was the NIRStar 14.2 (Version 14, Revision 2, Release Build, 2016-04-15 NIRx Medizintechnik GmbH, Berlin, Germany; www.nirx.net).

**Figure 1:**
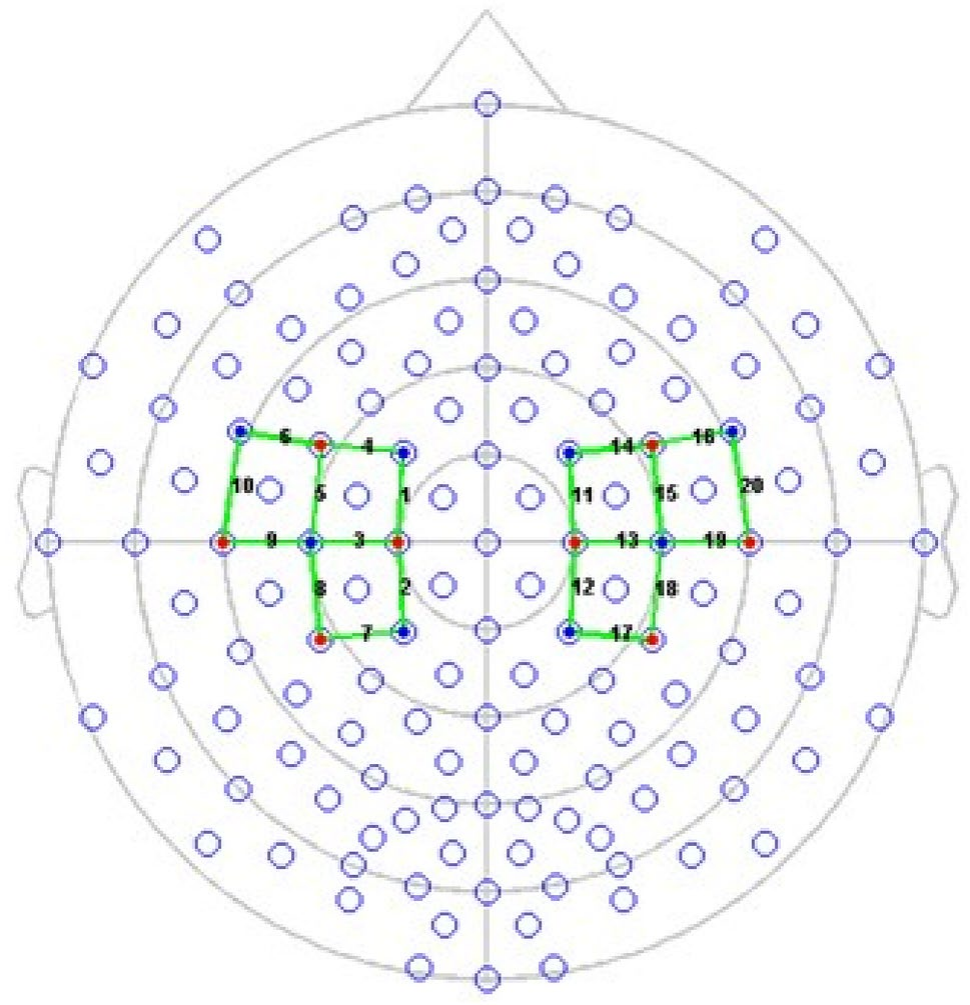
Channels and optodes configuration. Red circles indicate sources. Blue circles represent detectors. Green lines show the recording channels.

The fNIRS device included LED sources and photosensitive detectors (sensitivity: > 1 pW, dynamic range: > 50 dB). The researchers adopted eight transmitters (sources) for detecting changes in the hemodynamic activity, each transmitting two wavelengths, one being approximately 760 nm, whereas the other was found at approximately 850 nm. The sources and detectors arrangement covered a total of 20 fNIRS measurement channels, 10 for each side of the brain hemisphere (Fig. 1). The distance between light sources and detectors was 30 mm, as suggested in a previous work^2^. In particular, the optodes on the fNIRS cap were placed over the primary and supplementary motor cortex. Oxyhemoglobin (HbO_2_) and deoxyhemoglobin (Hb) concentrations were recorded at each location at a sampling rate of 7.81 Hz. A calibration procedure preceded each recording. During this procedure, the NIRSport instrument determined the signal amplification needed for each source-detector combination.

The NIRS channels were included in a multichannel EEG recordable cap with 65 electrodes. The present study did not take into account EEG data.

### Stimuli

**Figure 2:**
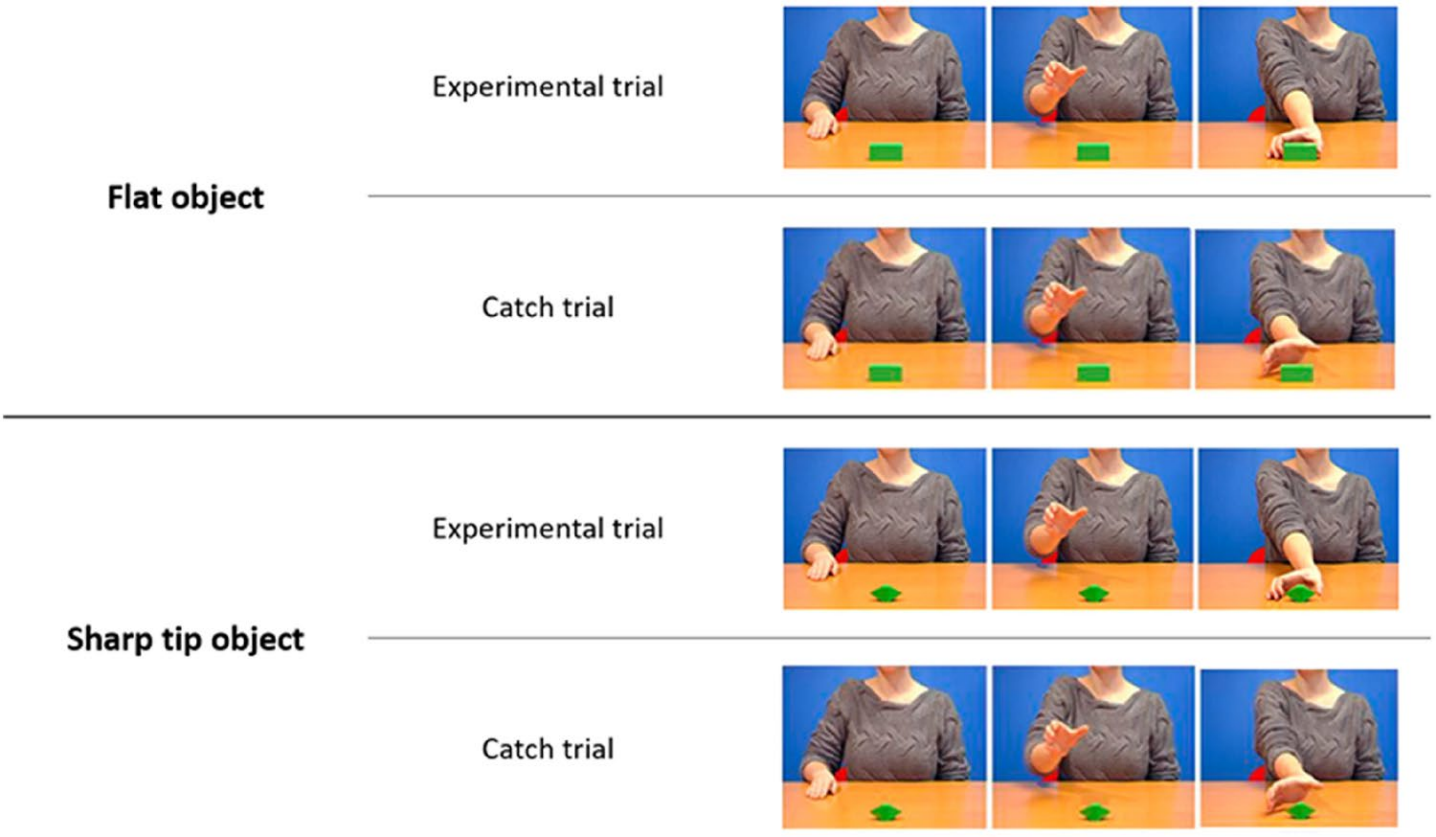
Three frames extracted from the flat object video (top) and the sharp-tip object video (bottom). Specifically, for each video, three frames extracted from the Experimental trial (Frame 1; Frame 25; Frame 66), and the Catch trial (Frame 1; Frame 25; Frame 38. Frame 38 was repeated 28 times to obtain the same duration as that of the experimental videos, 66 frames) are shown. The sharp-tip object videos were obtained by video editing the flat object videos. By means of a graphic software, the to-be-grasped parallelepiped was replaced by a polyhedron having the same size, but with sharp tips at the fingers opposition space.

Stimuli were the same as Craighero et al*.*^28^. They consisted in two experimental-trial videos and two catch-trial videos. In a third-person perspective, the experimental-trial videos showed an agent sitting at a desk making a movement for reaching and grasping one of two possible objects (Fig. 2). In the “flat object video” the object consisted of a parallelepiped, a square cuboid having 2 square and 4 rectangular faces (named “flat object”; width: 7 cm; height: 3 cm; length: 3 cm). The parallelepiped was placed with its longer axis facing the agent. The agent reached and grasped the parallelepiped with a natural velocity, with the fingers opposition space parallel to her frontal plane, without lifting the object. In the “sharp-tip object video”, using software for video editing, the parallelepiped was replaced with a polyhedron (i.e., a geometric solid in three dimensions with flat faces and straight edges) of the exact dimensions as the parallelepiped (named “sharp-tip object”; 7 cm × 3 cm × 3 cm). Thanks to this graphic trick, in the sharp-tip object video, the agent reached and grasped the polyhedron with her fingers precisely at the sharp tips, with the same kinematic parameters present in the flat object video. The two videos had the same time duration (2640 ms), and the instant at which the experimenter’s index finger touched the object was the same for both (1880 ms, Frame 47). It should be noted that the high weight of the object (240 g), and the presence of sharp tips right at the point of contact with the fingers, made it impossible to grasp the sharp-tip object with the grip shown in the video.

The two videos were further manipulated to obtain the two catch-trial videos in which the agent’s hand stopped before touching the objects (1520 ms after the beginning of the video, Frame 38). The last frame was repeatedly presented to obtain the same time duration as the experimental-trial videos, i.e., 2640 ms (Fig. 2). For more technical details, see Craighero et al*.*^28^. Catch-trial videos were introduced solely to force participants to constantly pay attention to video content and were never considered in the analysis of the results.

### Procedure

The experiment was conducted in a quiet room. The participants were seated, on a comfortable chair, in front of a table on which there were a monitor (placed 60 cm far from the participant) and a keyboard. Before starting the experiment, participants were asked to grab and lift the two objects once, one at a time, with the same grip which would be shown in the videos. This test allowed the participants to realize that the sharp-tip object could not be grasped with that specific grip as it caused pain. On the contrary, they realized that they were easily able to grab and lift the flat object.

Participants were submitted to two experimental sessions named Time-to-contact detection and Observation-only, respectively. Each session consisted of 42 trials randomly presented: 30 experimental trials (15 flat object videos, and 15 sharp-tip object videos) and 12 catch trials (6 flat object catch videos and 6 sharp-tip object catch videos). A black screen, lasting 15 s, was presented between one video and the subsequent. Before the presentation of the trials, each session included the recording of 100 s of resting state. Thus, each session included 5 conditions: Resting State, experimental trial of flat object video (Grasping Flat), experimental trial of sharp-tip object video (Grasping Sharp-tip), catch trial of flat object video and catch trial of sharp-tip object video.

Between the two experimental sessions, participants rested for 5 min. The sessions order was randomized among subjects. Furthermore, before the resting state recording, each session included the recording of 20 s of baseline, which was used in the signal processing phase. During both the baseline and the resting state, participants fixed a cross in the center of a black screen.

#### Time-to-contact detection session

Participants were instructed to watch the videos and press the space bar on the keyboard, with their right index finger, at the same instant the agent touched the to-be-reached object (experimental trials); conversely, they had to refrain from tapping the space bar when the agent’s hand stopped before touching the object (catch trials). The participant’s left arm was relaxed on the table. The number of catch trials to which the participant responded was noted. If it was equal to or greater than 6, the participant was discarded from this study. A low number of responses to catch trials ensured that the response was actually given at the moment of touch and not as the result of other clues.

#### Observation-only session

Both hands were relaxed on the table. Participants were asked to observe and pay attention to the videos. Six times, at the end of randomly selected trials, the following question appeared on the screen “what object did you just see?”. The participant answered the question verbally, and the researcher noted and verified the answer. If the number of errors was equal to or greater than 3, the participant was discarded from this study. A low number of errors ensured that the participant really paid attention to the videos.

### Data analysis and statistics

#### Behavioural data

The considered dependent variable was the time lag between the instant at which the agent’s index finger touched the object (*Instant of Touch*), i.e., 1880 ms from the beginning of each video, and the participant’s key pressing (*Response*). For each participant, for each trial, we calculated the time lag as *Instant of Touch—Response*. It should be noted that the purpose of the behavioural experiment was to verify the presence of a modulation of the response times according to the graspability of the object, as the modulation is considered an index of motor resonance. To this purpose, the time lag was submitted to an ANOVA, with Group (FM patients vs Controls) as between-subjects variable and Object (flat object vs sharp-tip object) as within-subject variable.

#### fNIRS data

The researcher used a custom-made script by nirsLAB, a commercial software MATLAB-based (nirsLAB, version 2017.06, NIRx Medical Technologies, Glen Head, NY, USA) to process fNIRS signals. Signal processing required several nirsLAB software functions: removing discontinuities from the signal, motion spike artefacts removal, baseline correction, and the molar extinction coefficients of hemoglobin.

Two independent researchers inspected the fNIRS signal to individuate the motion artefacts to be removed. The digital filter for the raw data recording was in the band-pass 0.06–0.2 Hz, allowing the removal of low oscillations from the fNIRS signal, such as respiratory and cardiac frequencies. The spectrum published by W.B. Gratzer (Med. Res. Council Labs, Holly Hill, London) and N. Kollias (Wellman Laboratories, Harvard Medical School, Boston, MA, USA) was selected for the molar extinction coefficients of hemoglobin.

Optical intensity measurements were converted to oxyhemoglobin (∆HbO_2_) and deoxyhemoglobin (∆Hb) concentration by using the modified Beer–Lambert law. The unit of measurement of hemoglobin concentration was mmol per liter (mmol/liter). To subtract the baseline, i.e., the first 20 s acquired for each recording, the corresponding range of timeframes was entered for each subject, and for the corresponding session, in the nirsLAB software before the application of the modified Beer–Lambert law.

The analysis also included the effect of the age-dependent DPF (Differential Path-length Factor), for which parameters were inserted in the software acquisition signal before starting each recording^3^. Then, for each experimental condition, the researcher computed the mean values of the hemodynamic concentration to detect specific changes in ∆HbO_2_ and ∆Hb during the task performance. The analysis window for each event was 8 s. The GLM approach was firstly used, for each subject during each task, to identify how the investigated brain areas activated. To do this, the hemodynamic response function (HRF) was performed to model the fNIRS signal during each single experimental session. SPM-1 within-subject analysis allowed estimating the activation (beta values) in each fNIRS channel with respect to the baseline^4^.

#### Statistical analysis

Statistical data analysis was performed using the IBM SPSS Statistics software, version 21. For all the statistical tests, a *p*-value lower than 0.05 was considered statistically significant.

*Behavioural data* For the time lags measured during sharp-tip object and flat object videos, we applied two ways ANOVA with groups (FM patients vs Controls) as between-subjects variable, and object (sharp-tip object vs flat object) as within-subject variable.

*fNIRS data* We computed a MANOVA analysis separately for oxyhemoglobin and deoxyhemoglobin levels change. The 20 fNIRS channels were the independent variables, whereas the group (FM patients vs Controls), the condition (Resting State vs Grasping Flat vs Grasping Sharp-tip) and session (Time-to-contact detection vs Observation only) were the factors. A post-hoc Bonferroni test was applied to compare oxyhemoglobin and deoxyhemoglobin levels among conditions. The Roy square was considered.

To obtain the graphical representation of the brain activity in accordance with the statistical analysis, the SPM-2 analysis was performed as a between-subjects analysis to find the fNIRS channels with significant variations between groups about ∆HbO_2_ and ∆Hb concentrations. The software for performing fNIRS topographical analysis was the Statistical Parameter Mapping NIRS-SPM (SPM 8) tool executed in NIRSlab (version 2017.6).

## Results

### Behavioural data

Responses given during catch trials were almost absent. In particular, 5 FM patients gave one response, whereas the others did not give any response to catch trials. Regarding control subjects, 3 of them gave one response to catch trials, whereas the others did not give any response to catch trials. None of the participants ever made any mistake answering the question related to the identity of the object presented in the previous trial.

**Figure 3:**
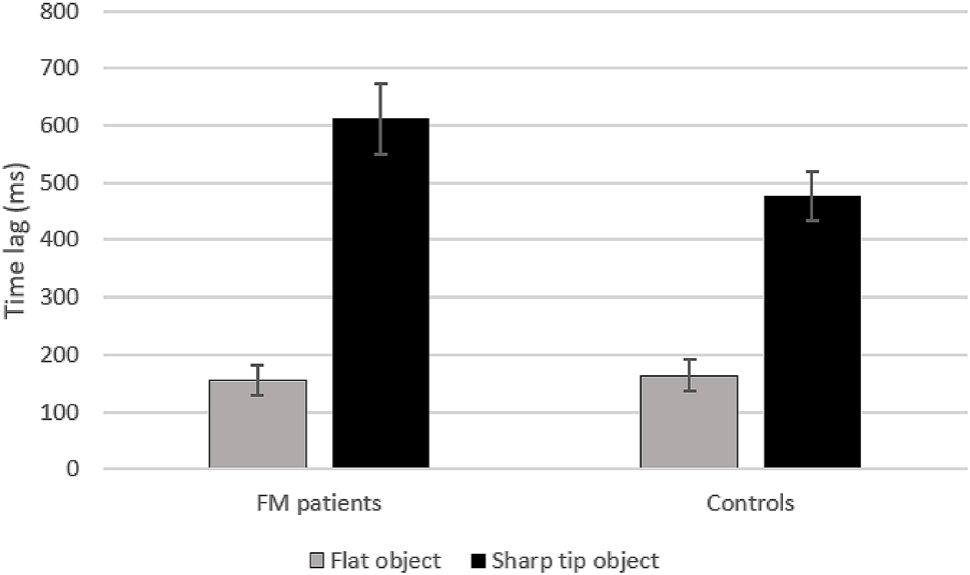
Detection time results. Time lag between the instant at which the agent touches the object and participant’s response time. For both groups (FM patients, Controls), data for flat object trials (grey) and sharp-tip object (black) trials are shown. Thin lines above histograms indicate standard error of the mean. Ordinates are in milliseconds.

The results of the ANOVA performed on the time lag showed that the object main effect was significant (*F*_1,40_ = 95.409, *p* = 0.000, *ηp*^2^ = 0.705) since the time lag during sharp-tip object trials (mean = 547.34 ms, SEM = 39.38) was longer than during flat object trials (mean = 159.37 ms, SEM = 19.20). The group main effect (*F*_1,40_ = 1.834, *p* = 0.183, *ηp*^2^ = 0.044), and the 2-way interaction group × object (*F*_1,40_ = 3.375, *p* = 0.074, *ηp*^2^ = 0.078) were not significant. The detection time results were reported in Fig. 3.

Results indicate that both FM patients and controls show a modulation of the response times according to the graspability of the object. Therefore, behavioral data suggest that motor resonance is present in both groups.

### fNIRS-oxyhemoglobin

The results of MANOVA analsysis performed on the fNIRS oxyhemoglobin data, among conditions (Resting State vs Grasping Flat vs Grasping Sharp-tip), session (Time-to-contact detection vs Observation-only) and groups (FM patients vs controls) are reported in Table 1.

**Table 1:** MANOVA results.

Variable	*F*	*DF*	Error *df*	Sig
Group	3.857	20	209	< 0.001
Condition	1.108	20	210	0.34
Session	3.128	20	209	< 0.001
Group × condition	4.042	20	210	< 0.001
Group × session	2.575	20	209	< 0.01
Condition × session	3.262	20	210	< 0.001
Group × condition × session	2.083	20	210	< 0.01

Dependent variable: 20 fNIRS channels for oxyhemoglobin level, Between subjects’ factor: Condition (Resting State vs Flat object vs Sharp-tip object), and Session (Time-to-contact detection vs Observation-only) as within-subject factors, and Group (FM patients vs Controls). The Roy square was considered.

**Figure 4:**
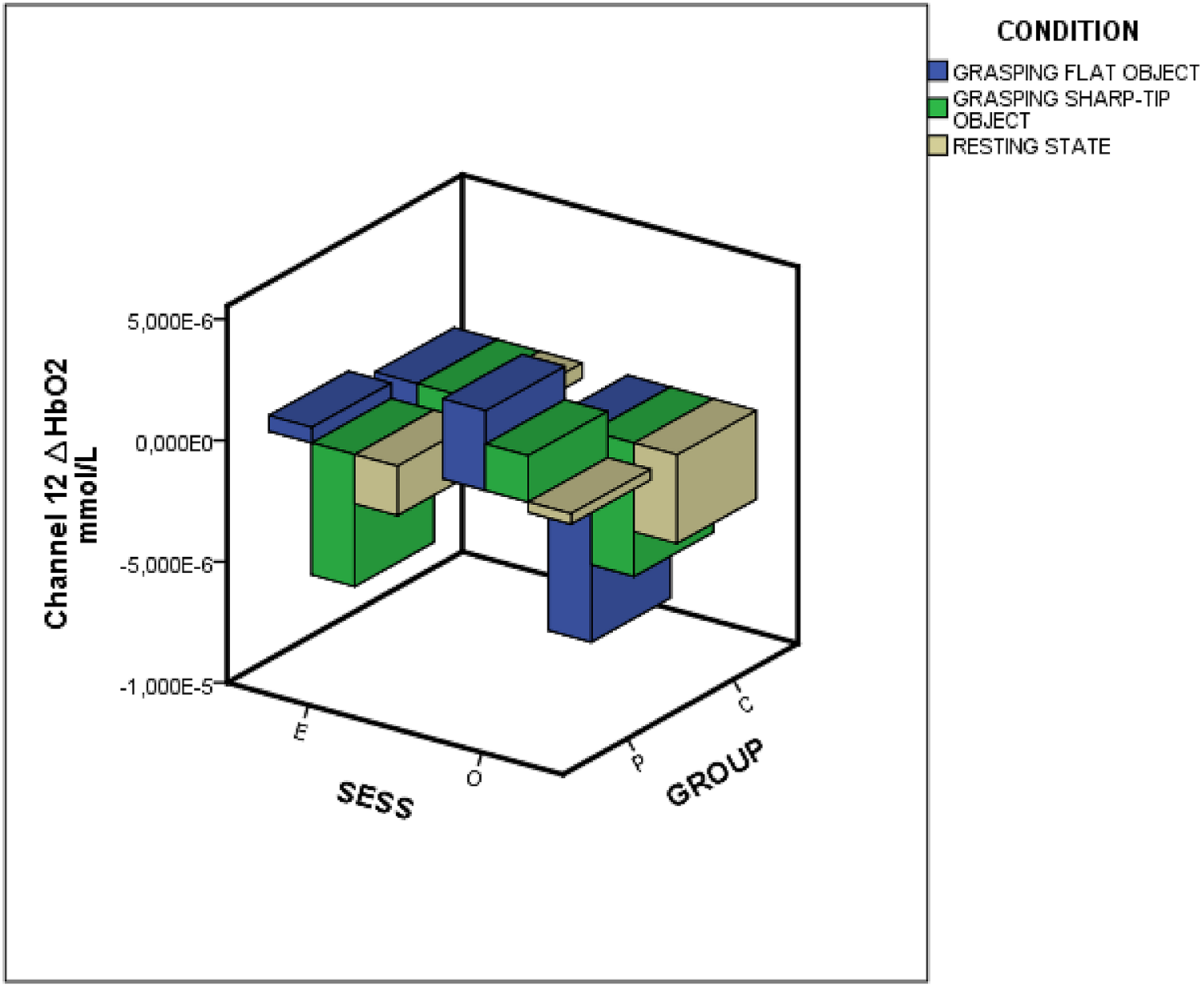
Oxyhemoglobin levels on an exemplificative channel (channel 12). *Sess* session, *O* observation-only session, *E* time-to-contact detection session, *P* FM patients, *C* controls.

**Figure 5:**
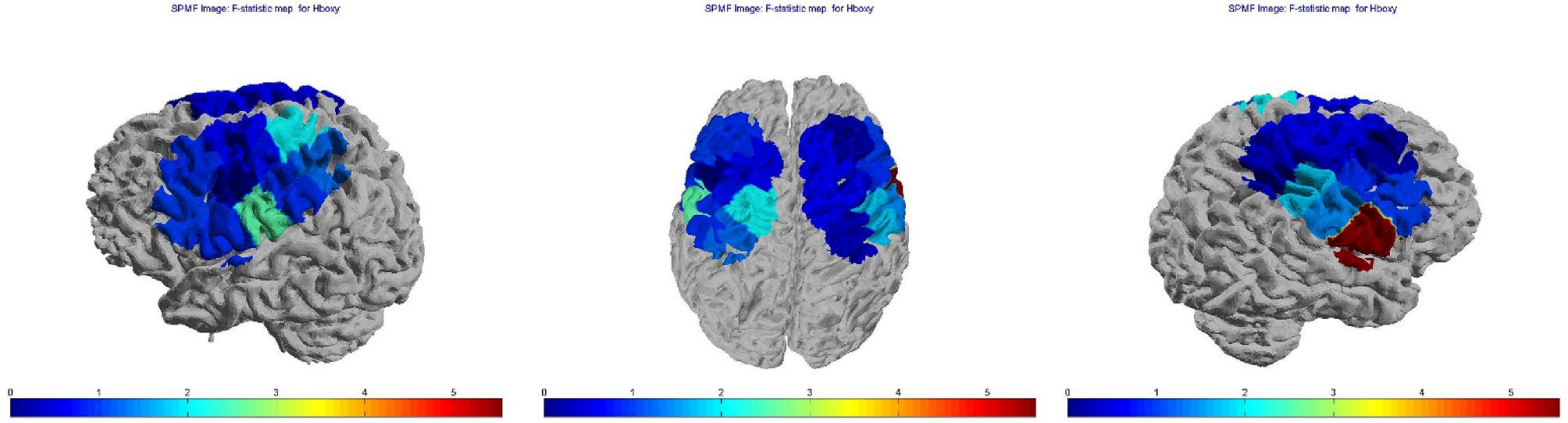
Topographic maps of F-statistic in the comparison among conditions (Resting State vs Grasping Flat vs Grasping Sharp-tip object), session (Time-to-contact detection vs Observation-only) and groups (FM patients vs controls). Blue areas represent channels with no significant change in hemoglobin levels, red areas represent channels where the variations in hemoglobin levels were significant.

In detail, the within-channels analysis (20 channels) showed statistical differences as effect of group, session, and the interactions group × condition, group × session and group × session × condition (Fig. 4). The interaction group ·× condition × session was significant on channel 20 (*p* < 0.01). Figure 5 shows the *F*-statistic for hemoglobin of all the channels evaluated on the group × condition × session interaction.

**Figure 6:**
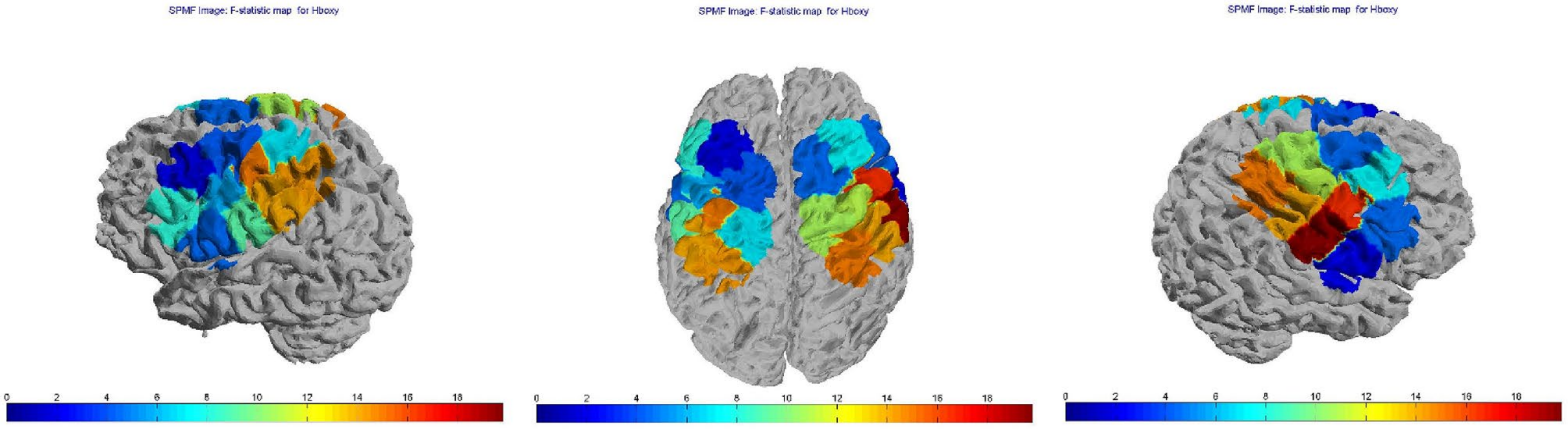
Topographic maps of F-statistic in the comparison among session (Time-to-contact detection vs Observation-only) and groups (FM patients vs controls). Blue areas represent channels with no significant change in hemoglobin levels, red areas represent channels where the variations in hemoglobin levels were significant.

**Figure 7:**
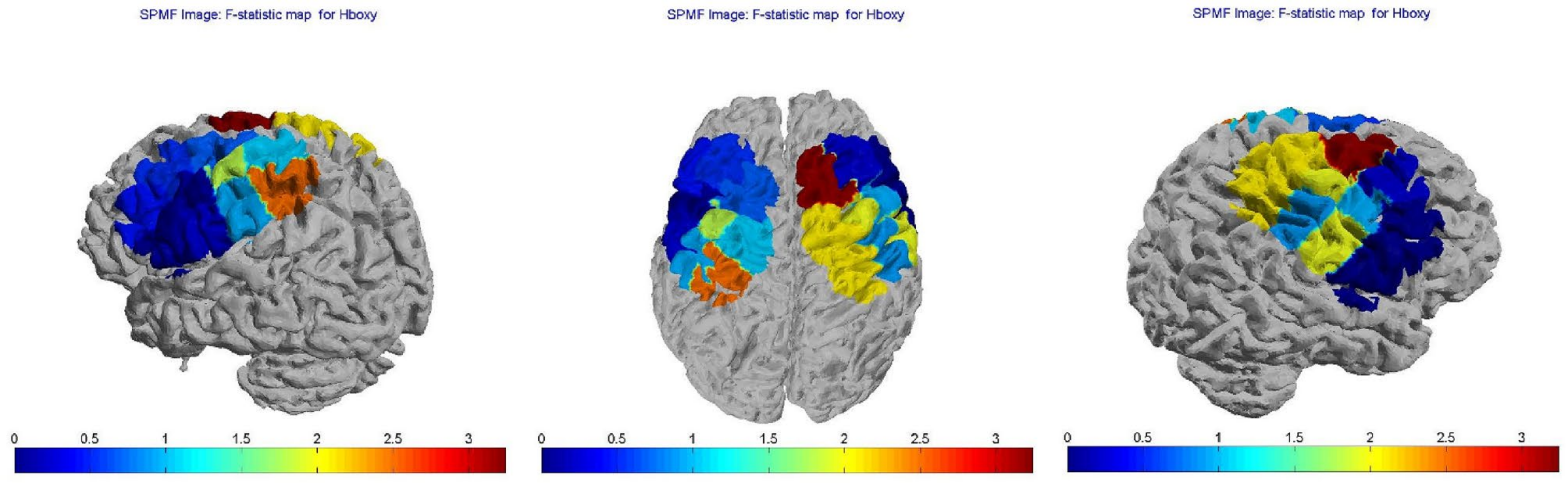
Topographic maps of F-statistic in the comparison among groups (FM patients vs controls) and conditions (Resting State vs Grasping Flat vs Grasping Sharp-tip object). Blue areas represent channels with no significant change in hemoglobin levels, red areas represent channels where the variations in hemoglobin levels were significant.

The interaction group × session was significant on all channels (*p* < 0.01), except for channels 4 and 20 (channel 4, *p* = 0.079; channel 20, *p* = 0.08). Figure 6 shows the *F*-statistic for hemoglobin of all the channels evaluated on the group × session interaction. The interaction group × condition was significant on channel 12 (*p* = 0.048). Figure 7 shows the *F*-statistic for hemoglogin of all the channels evaluated on the group × condition interaction.

**Figure 8:**
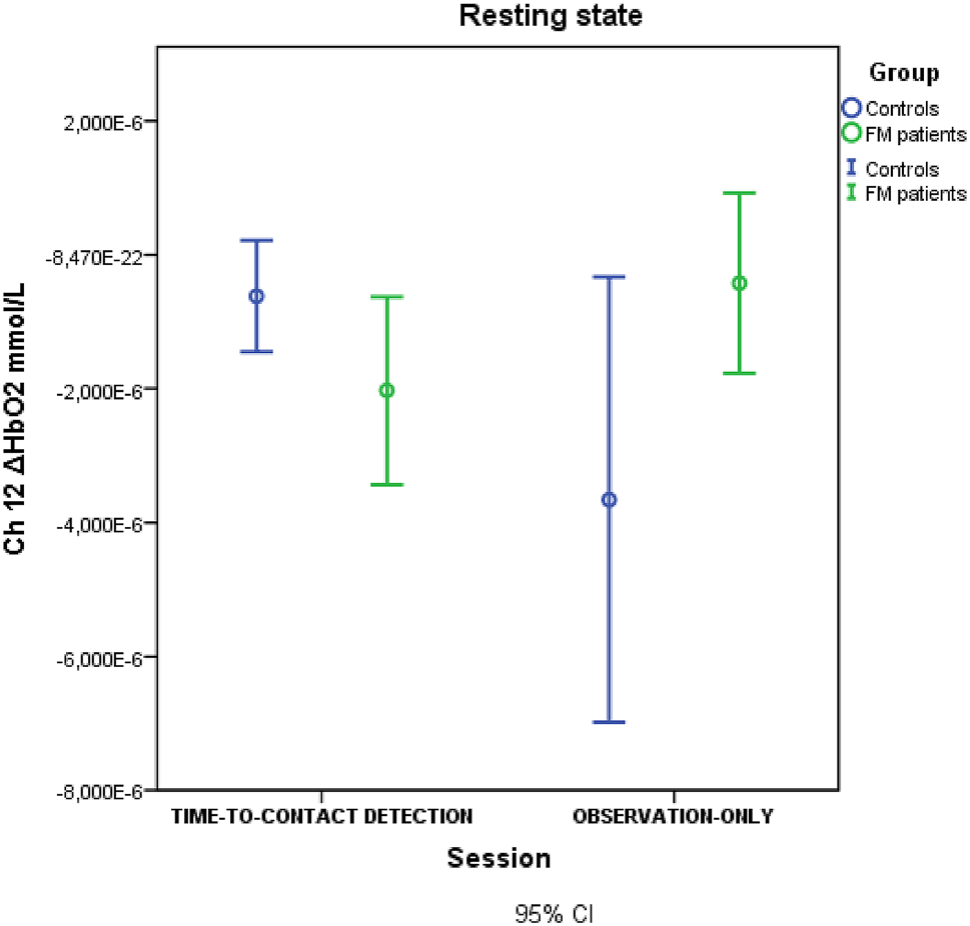
Mean and 95% CI of oxyhemoglobin levels recorded during the resting state preceding the time-to-contact detection session and the resting state preceding the observation-only session in a representative channel (channel 12).

Considering the Resting State condition preceding each session, we observed an increase in oxyhemoglobin levels for FM patients before the Observation-only session in channels 3, 6, 8, 9, 12, 13, 15, 16, 17, 18, and 19 (session: *F* = 0.88, *p* = 0.6; group: *F* = 2.58, *p* = 0.03; session × group *F* = 1.88, *p* = 0.033). Figure 8 shows, for channel 12, such increment.

Since the group factor was statistically significant, the results were analysed separately for FM patients and Controls.

Manova analyses revealed that control subjects showed an increase of oxyhemoglobin levels during the Time-to-contact detection session which was present in all channels (*p* < 0.05), except for channels 4, 10 and 20. The interaction session × condition was significant on channel 19 (*p* < 0.05). The Bonferroni test among conditions was not significant (Table 2).

**Table 2:** MANOVA results in controls.

Variable	*F*	*DF*	Error *DF*	Sig
Session	3.590	20.00	92.00	< 0.001
Condition	0.797	20.00	93.00	0.71
Session × condition	1.660	20.00	93.00	0.05

Dependent variable: 20 fNIRS channels for oxyhemoglobin level, factors: Condition (Resting State vs Grasping Flat vs Grasping Sharp-tip object) and Session (Time-to-contact detection vs Observation-only).

**Figure 9:**
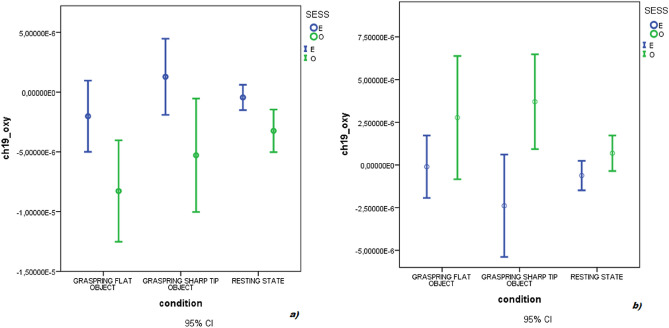
Mean and 95% CI of oxyhemoglobin level in a representative channel (19) in controls (**a**) and FM patients (**b**). During the Time-to-contact detection task, oxyhemoglobin increased in controls and decreased in FM. In FM patients, the decrease of oxyhemoglobin level during this session was specifically present during sharp tip object trials.

**Figure 10:**
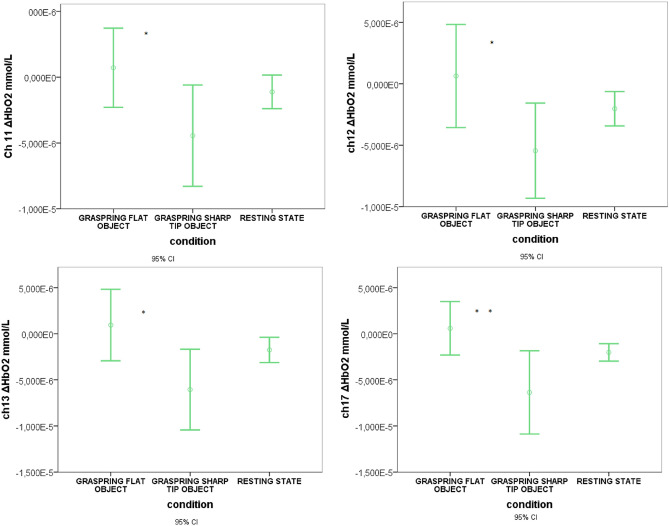
Mean and 95% CI of oxyhemoglobin levels recorded during the Time-to-contact detection session in FM patients. The results of Bonferroni tests are reported. Comparison between conditions Grasping Flat versus Grasping Sharp-tip object: *p < 0.05; **p < 0.001. The oxyhemoglobin level was significantly lower during sharp-tip object trials than during flat object trials. The comparisons with the Resting State did not reach the statistical significance, so the main phenomenon we observed was the contrast between cortical activation modality in grasping flat vs grasping sharp-tip object.

On the contrary, the analysis revealed, for FM patients, a decrease of oxyhemoglobin levels during the Time-to-contact detection session, whereas the factor condition and the interaction condition × session were not statistically significant (Table 3). The oxyhemoglobin increased significantly during the Observation-only session on channels 3, 13, 15, 18 (*p* < 0.05) and channels 6, 7, 8, 12, 17, 19 (*p* < 0.01) as shown in Fig. 9. The interaction session × condition was significant on channels 8, 13, 15, 17, 19, 20 (*p* < 0.05), as shown in Fig. 10. In fact, the oxyhemoglobin levels increased during the flat object grasping vision only during the Time-to-contact detection session.

**Table 3:** MANOVA results in FM patients.

Variable	*F*	*DF*	Error *DF*	Sig
Condition	1.438	20.00	99.00	0.12
Session	2.154	20.00	98.00	0.01
Condition × session	1.467	20.00	99.00	0.11

Dependent variable: 20 fNIRS channels for oxyhemoglobin level, factors: Condition (Resting State vs Grasping Flat vs Grasping Sharp-tip object) and Session (Time-to-contact detection vs Observation-only).

A Bonferroni post-hoc analysis was conducted on the three conditions (Resting state vs Grasping Flat vs Grasping Sharp-tip object). The comparisons, in each channel, with the Resting State did not reach the statistical significance, so the main phenomenon we observed was the contrast between cortical activation modality in Grasping Flat versus Grasping Sharp-tip object.

### FNIRS-deoxyhemoglobin

**Figure 11:**
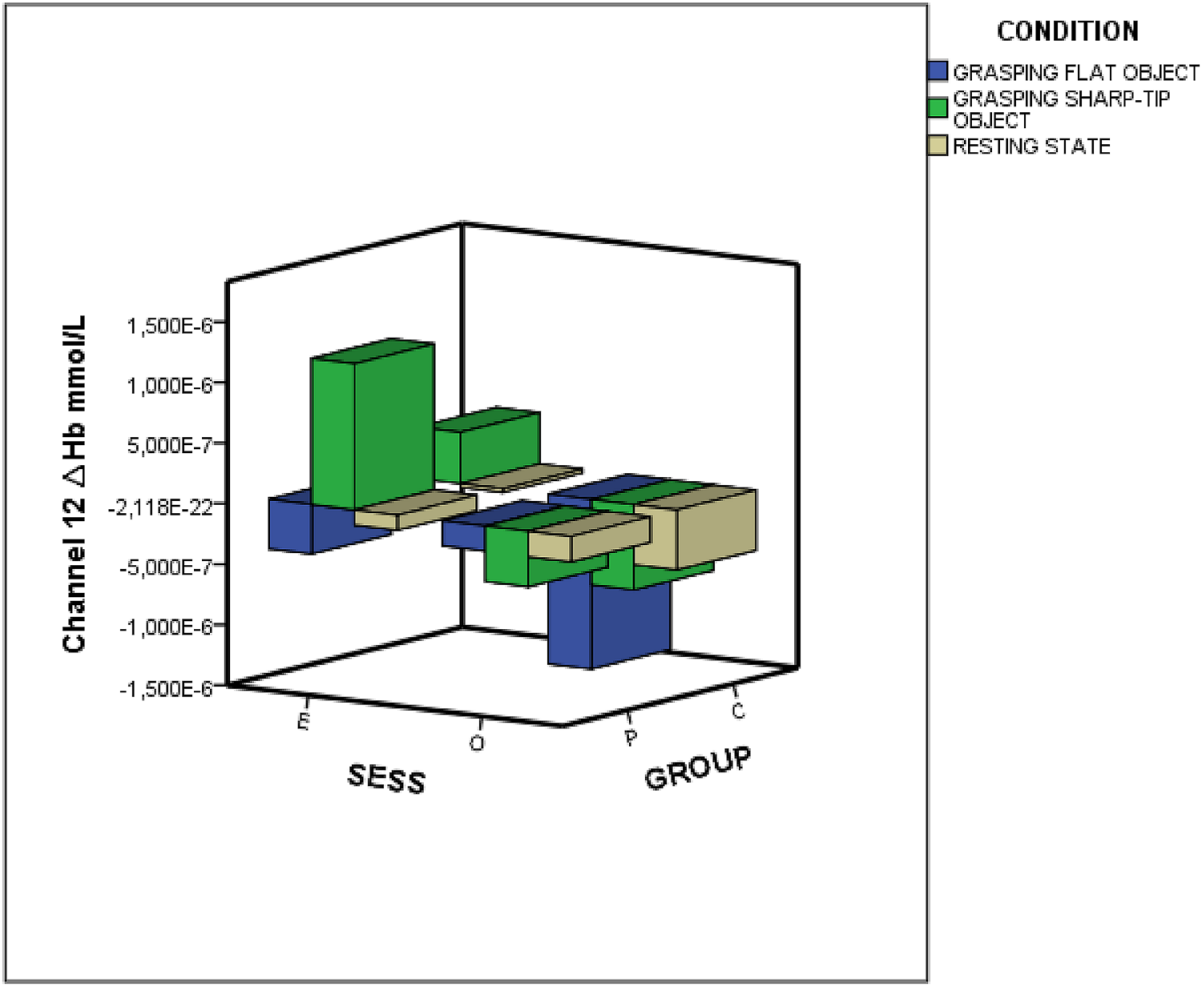
Deoxyhemoglobin levels on an exemplificative channel (channel 12). *Sess* session, *O* observation-only session, *E* time-to-contact detection session, *P* FM patients, *C* controls.

Deoxyhemoglobin levels were significantly different between the Time-to-contact detection and the Observation-only sessions (Table 4). The deoxyhemoglobin levels decreased during the Observation-only sessions and tended to increase during the Time-to-contact detection session (Fig. 11). No other factors or interactions were significant.

**Table 4:** MANOVA results.

Variable	*F*	*DF*	Error *DF*	Sig
Group	1.071	20.00	209.00	0.38
Session	5.142	20.00	209.00	< 0.001
Condition	0.122	20.00	210.00	1.00
Group × session	0.017	20.00	209.00	1.00
Group × condition	0.115	20.00	210.00	1.00
Session × condition	0.088	20.00	210.00	1.00
Group × session × condition	0.108	20.00	210.00	1.00

Dependent variable: 20 fNIRS channels for deoxyhemoglobin level, factors: Condition (Resting state vs Grasping Flat vs Grasping Sharp-tip object), and Session (Time-to-contact detection vs Observation-only) and Group (FM patients vs Controls). The Roy square was considered.

## Discussion

For about 30 years, it has been known that action observation determines a modulation of the sensorimotor system, named motor resonance^1^. However, it has recently been found that this modulation is influenced by top-down information based on context, stimulus characteristics, and task requirements^25^. Among the possible factors capable of inhibiting motor resonance, there is the knowledge that the observed action causes pain to another individual^33–35^. One possible interpretation could be that the observation of pain automatically induces hidden simulation of potentially adaptive freezing and avoidance responses in the viewer’s corticospinal system^33,34^. This rare situation in the healthy population appears to be the norm in patients with FM, a chronic pain disease characterized by generalized musculoskeletal pain^16^. In fact, in these patients, a simple movement triggers an exacerbation of symptoms that leads to fear of pain and consequently to avoidance behaviors towards movement^22^. Therefore, it is possible that, in FM patients, the disease state can affect sensorimotor activation not only when observing a grasping action that causes pain but also when observing a simple grasping movement.

To test this possibility, the present study investigated at a behavioral and neurophysiological level the presence of motor resonance in FM patients and compared their results with those of healthy controls. To this purpose, participants were submitted to a behavioral task known to reveal the presence of motor resonance^28,30,36,37^. Specifically, they were presented with videos showing grasping movements towards a graspable (i.e., flat object) or an ungraspable (i.e., sharp tip object) object, and were instructed to press a key at the instant at which the agent touched the object (i.e., Time-to-contact detection session). Note that grasping the sharp-tip object was impossible as it was painful. The greater accuracy in response to graspable object trials is considered indirect proof of the presence of motor resonance^28^. Furthermore, in a different session, participants were only requested to observe and pay attention to the videos (i.e., Observation-only session). Previous results^28^ showed an increase in corticospinal excitability during observation of graspable objects trials. On the contrary, the corticospinal excitability when observing ungraspable objects trials was not different from that recorded during the control condition. This result suggests that the motor system of the observer is suppressed/inhibited during observation of unsuitable actions. During both experimental sessions, the hemodynamic activity of the participants was recorded by fNIRS. We were interested in verifying whether in FM patients the observation of actions is able to activate the motor cortex, given that previous studies in these patients^31^ showed that the motor cortex exhibits reduced levels of oxyhemoglobin during the resting state.

### Behavioral results

Behavioral results revealed that response times in patients did not differ from those of healthy participants. Specifically, in both groups, response times were modulated by the presence of objects with different intrinsic properties. Responses were more accurate (i.e., shorter time lag between the instant at which the agent’s touched the object and the participant’s key pressing) during flat object trials than during sharp tip object trials. This result is congruent with those of previous studies in which the same experimental protocol was used^28,30,36,37^. These findings could be considered evidence that during action observation the individuals automatically activate the relative sensorimotor representation built on their own experience. Therefore, when observing actions that the individual would never perform, the motor system is inhibited, as suggested by the absence of modulation in corticospinal excitability^28,35^. The lack of motor simulation prevents the same level of accuracy in time-to-contact detection that occurs when observing an action that we would have no problem performing. Thus, behavioral results suggest that motor resonance is present in both groups.

Future studies will aim to standardize this experimental protocol to have the possibility to investigate the effects at the individual level and develop efficient treatments in function of interindividual variability.

### fNIRS results

During the resting state preceding the observation-only session Fm patients showed a cortical activation which was not present in controls, while before the Time-to-contact detection session, patients had lower levels of oxyhemoglobin in respect to controls. Therefore, in FM patients, the observation of the other’s movement properties could represent a compensation process for their basal motor insufficiency, which could emerge in the preparation for active movement. In general, the patients' cortical motor activation was congruent with the intrinsic properties of the graspable objects, increasing with the observation of videos reproducing movement toward the flat tool. In FM patients, the vision of objects with different intrinsic properties modulated cortical metabolism together with response times.

In controls, the observation of movements to the flat object tended to reduce cortical metabolism, with an opposite tendency toward metabolism increase during sharp-tip object grasping. These results were not statistically significant but were suggestive of a possible motor network activation in the attempt to correct other’s movement incongruence.

In previous studies in this field, TMS demonstrated that the excitability of the motor cortex changed in agreement with the characteristics of the observed movement^28^. The two measures are fundamentally different, and describe cortical phenomena in a different way, at different times, and in different brain areas. Transient activation of the primary motor cortex, as indicated by TMS, could be followed by further phenomena in supplementary motor areas, mapped by fNIRS. As a matter of fact, fNIRS reports the global changes in activation of the primary motor networks, including phenomena of motor planning integration. This means that the global tendency of motor networks in healthy subjects is toward a possible activation able to correct the observed incongruent movement. In mild motor networks failure, as that present in FM patients^31^, cortical metabolism is trained by the observed movement properties.

In a previous study using the fNIRS method, the motor-only imagery generated a moderate activation in primary motor cortex activity similar to that of motor execution in healthy subjects^38^. Motor imagery is a mental function by which an individual experiences a given action, thanks to which motor networks could reproduce and prepare a correct kinematic^39^. Our study could just demonstrate that during the observation of others’ movement, the normal cortical networks could be activated in a direction of a possible others’ movement interaction and correction.

In the topographic analysis of oxyhemoglobin changes, we observed a bilateral cortical modulation with increased activation of motor networks in FM patients during the observation of the congruent movement with the intrinsic characteristics of the object.

Bilateral activation of the premotor cortex has been observed in healthy subjects during tasks employing action observation^40^. M﻿oreove﻿r, in patients with motor impairment, the contralateral motor networks were activated during the hand movement observation^41^. In FM patients, the observation of congruent movement executed with the right hand determined a compensation of basal hypometabolism with prevalent recruitment of the contralateral supplementary motor areas.

The fundamental cortical resources of FM patients could thus be activated during motor observation, as a compensatory phenomenon to pain-related motor impairment.

### Study limitations

There is still uncertainty regarding fNIRS parameters and results. We evaluated both oxyhemoglobin and deoxyhemoglobin, observing an opposite behavior. However, significant results attained only the oxyhemoglobin, so the relevance of deoxyhemoglobin parameters still needs confirmation. The time lag of metabolic changes is long and could just include different cortical areas, M1, ipsilateral, and contralateral supplementary motor cortex, without the possibility to disentangle single cortical regions activation. The maximal peak of oxyhemoglobin change is variable among subjects, with a possible cutting off of maximal values in single cases^42^. However, the time analysis of 8 s enabled a good compliance for individual responses.

## Conclusions

Present findings extend current knowledge regarding cortical motor activation functioning during the execution or observation of action in a specific group of patients with chronic pain. Motor performance is impaired in FM patients^31^, and motor cortex metabolism basically reduced when strictly related to active movement. Movement observation provokes motor networks activations and modulation, which suggests cortical adaptation mechanisms able to restart a virtuous phase of beneficial interaction with pain-related circuits^43^.

By virtue of the effective modulation role on pain played by the activation of the motor cortex^44^, this study can represent a useful contribution to the programming and optimization of rehabilitative motor protocols, like those included in AOT, that are less tiring and more involving for patients with fibromyalgia than physical exercise.
